# An in silico analysis of *rpoB* mutations to affect *Chlamydia trachomatis* sensitivity to rifamycin

**DOI:** 10.1186/s43141-022-00428-y

**Published:** 2022-10-25

**Authors:** Ichrak Benamri, Maryame Azzouzi, Ahmed Moussa, Fouzia Radouani

**Affiliations:** 1 Chlamydiae and Mycoplasma Laboratory, Institut Pasteur du Maroc, 20360 Casablanca, Morocco; 2 Systems and Data Engineering Team, National School of Applied Sciences, Abdelmalek Essaâdi University, BP1818, Route Ziaten, Tangier, 90 000 Morocco; 3 Laboratory of Microbiology, Pharmacology, Biotechnology and Environment, Faculty of sciences Aîn-Chock, Hassan II University, Casablanca, Morocco

**Keywords:** *Chlamydia trachomatis*, *rpoB* gene, Mutations, In silico analysis, Antibiotic, Resistance

## Abstract

**Background:**

C*hlamydia trachomatis* is an obligate intracellular gram-negative pathogen, responsible for diverse affections, mainly trachoma and sexually transmitted diseases. Antibiotics are the commonly used drugs to tackle chlamydiae infections. However, when overused or wrongly used this may lead to strains’ resistance to antibiotics, this phenomenon represents a real health problem worldwide. Numerous studies showed the association of *Chlamydia trachomatis* resistance with mutations in different genes; these mutations could have a deleterious or neutral impacts on the encoded proteins. The aim of this study is to perform an in silico analysis of *C. trachomatis rpoB*-encoded proteins using numerous bioinformatics tools and to identify the functional and structural-related effects of the mutations and consequently their impact on the bacteria sensitivity to antibiotics.

**Results:**

The analysis revealed that the prediction of the damaging impact related to the mutations in *rpoB*-encoded proteins showed eight mutations: V136F, Q458K, V466A, A467T, H471N, H471Y, H471L, and I517M with big deleterious effects. Among them, six mutations, V136F, Q458K, V466A, A467T, H471N, and I517M, are located in a highly conserved regions decreasing the protein’s stability. Furthermore, the structures analysis showed that the mutations A467T, H471N, I517M, and V136F models had a high deviation compared to the wild type. Moreover, the prediction of protein-protein network indicated that *rpoB* wild type interacts strongly with 10 proteins of *C. trachomatis*, which are playing different roles at different levels.

**Conclusion:**

As conclusion, the present study revealed that the changes observed in the encoded proteins can affect their functions and structures, in addition to their interactions with other proteins which impact the bacteria sensitivity to antibiotics. Consequently, the information revealed through this in silico analysis would be useful for deeper exploration to understand the mechanisms of *C. trachomatis* resistance and enable managing the infection to avoid its complications. We recommend further investigations and perform deeper experimental analysis with collaboration between bioinformaticians, physicians, biologists, pharmacists, and chemistry and biochemistry scientists.

## Background

The family Chlamydiaceae comprises a group of obligate intracellular Gram-negative bacteria that are responsible for a broad range of infections in mammals, birds, and humans [1]. Current classification within this family recognizes a single genus within this family, *Chlamydia*, 14 species [2–4] and four *Candidatus* (Ca.) species (Ca. Chlamydia ibidis, Ca. Chlamydia corallus, Ca. Chlamydia sanzinia, and Ca. Chlamydia testudinis) [5–8]. Humans are only *Chlamydia trachomatis* (*C. trachomatis*) natural hosts [9]; when *C. trachomatis* bacteria is untreated, it may lead to severe complications. In females, pelvic inflammatory disease leads to tubal infertility [10–13], ectopic pregnancy [13], and chronic pelvic pain [14]. Furthermore, *C. trachomatis* can be transferred to the newborn from the infected mothers; they can develop ocular, respiratory, and gastrointestinal infections [15]. In men, it would be urethritis, epididymitis, prostatitis, and proctitis [16, 17]. In addition, lymphogranuloma venereum [18] and reactive arthritis [19] are the less common diseases caused by *C*. *trachomatis* in both men and women infected with *C. trachomatis*. In another hand, *C*. *trachomatis* produces chronic ocular infections that can lead to trachoma, which is one of the leading causes of blindness worldwide [20, 21].

The recommended treatment for chlamydia infections is antibiotics. However, the misuse or the overuse of these antibiotics may lead to antibiotic resistance. The acquired resistance occurs when the bacterium that has been sensitive to antibiotics develops resistance via mutation or via acquisition of new DNA [22].

Different studies showed that *C. trachomatis* may develop resistance to macrolides via mutations in the *23S rRNA* gene, to fluoroquinolones via mutations in the *gyrA* gene, and to rifamycins via mutations in the *rpoB* gene [20, 21, 23–27]. The resistance to rifamycins was shown to be associated with a nucleotide substitution in *rpoB* gene, impacting the inhibition of bacterial transcription related to interacting with beta-subunit of bacterial DNA-dependent RNA polymerase [28].

All mutations within the genomic sequence can lead to alterations in the sequence of the encoded protein, which could have a deleterious or neutral impacts on the protein. Furthermore, it may ultimately affect alteration of protein charge, geometry, hydrophobicity dynamics, translation, and inter- or intra-protein interaction set cells in danger [29].

The aim of the present study is to perform an in silico analysis of the retrieved amino acid variations in *C. trachomatis rpoB* gene-encoded protein and identify the functional and structural-related effects of the protein’s variations, which consequently may impacting the bacteria sensitivity to antibiotics.

## Methods

**Fig. 1:**
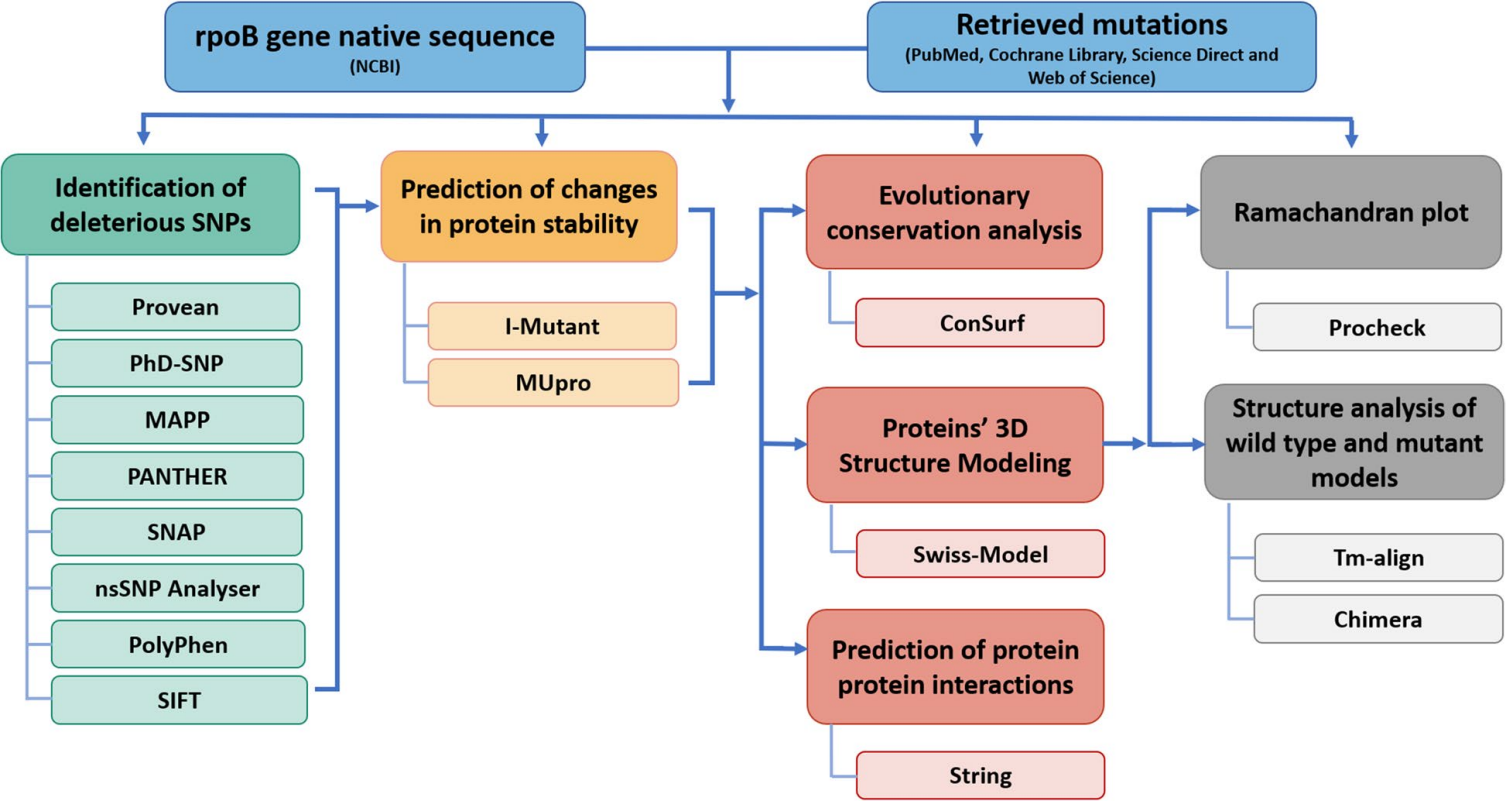
Study steps flowchart

In the present study, we performed an in silico analysis using different machine learning algorithms, following the various steps described below and illustrated in the flowchart (Fig. 1).

### Mutations collection

To find the *rpoB* gene mutations linked to *C. trachomatis* resistance to antibiotics, we proceeded to extract all the *rpoB* mutations from various resources, which were gathered in our precedent investigations [22].

### Prediction of *rpoB* mutations’ deleterious effects

The damaging effects of the mutations on the protein were predicted using PredictSNP1.0 (http://loschmidt.chemi.muni.cz/predictsnp1/) [30]; this tool includes nine different Bioinformatics’ tools: SIFT [31], PolyPhen-1 [32], PolyPhen-2 [33], MAPP [34], PhD-SNP [35], SNAP [36], PANTHER [37], PredictSNP [38], and nsSNPAnalyzer [39]. Most of these tools are designed to predict whether a particular substitution is neutral or deleterious, based on various parameters derived from the evolutionary, physicochemical, or structural characteristics.

PredictSNP1.0 displays the confidence scores generated by each tool and a consensus prediction as percentages by using their observed accuracy values to simplify comparisons [38]. We classified the mutations as deleterious if the results of five among the nine tools is identified as damaging.

### Prediction of changes on the protein stability

To predict the change on the protein stability, we performed the Sanavia et al. protocol, where the effects of the variants on the protein stability are quantified in terms of the Gibbs free energy of unfolding (ΔG), and the measure of interest is the difference of the unfolding free energy between the mutant and wild type proteins (ΔΔG*u*); the sign of ΔΔG indicates if the mutation decreases (ΔΔG*u* < 0) or increases (ΔΔG*u* > 0) the protein stability [40].

We analyzed the different approaches available and selected I-Mutant 3.0 and MUpro to perform this analysis. I-Mutant 3.0 (http://gpcr2.biocomp.unibo.it/cgi/predictors/I-Mutant3.0/I-Mutant3.0.cgi) is a support vector machine (SVM) and a web-based tool that provides the predicted change in Gibbs free energy (ΔΔG) [41]. MUpro server (http://mupro.proteomics.ics.uci.edu/) is based on SVM [38]. We submitted the input data of the retrieved mutations of *rpoB* in FASTA format.

### Evolutionary conservation analysis

The evolutionary conservation analysis consists in estimating the degree of the amino acid conservation based on multiple sequence alignment using the ConSurf server (https://consurf.tau.ac.il) [42]. The degree to which an amino acid position is evolutionarily conserved is strongly dependent on its structural and functional importance [42, 43]. In ConSurf, the evolutionary rate is estimated based on the evolutionary relatedness between the protein and its homologs and considering the similarity between amino acids as reflected in the substitutions matrix [44, 45]. In the present study, the homologous sequences were collected using BLAST (or PSI-BLAST) search against a selected database.

The grade ranges from 1 to 9 estimates the extent of conservation of the amino acid throughout evolution. Therefore, grade 9 represents the most highly conserved residue, and the numbers descend to 1 representing the least conserved region.

### Proteins’ 3D structure modeling and validation

To understand the effect of each mutation on the protein structure, the 3D models of *rpoB*-encoded protein and its selected mutants are designed with SWISS-MODEL (https://swissmodel.expasy.org/) [46]. According to QMEAN score, and sequence identity, the best quality model was selected. Furthermore, to confirm the models, the Ramachandran plots were generated with PROCHECK [47].

### Structure analysis of wild type and mutant models

To compare the native and mutated protein structures, structural similarities were calculated using TM-align tool (https://zhanglab.ccmb.med.umich.edu/TM-align/), based on template modeling score (TM-score) and the root mean square deviation (RMSD) scores [48]. Tm-align produces a result between 0 and 1. TM-score equal to 1 means that there is no difference between wild type and the mutant structure; however, TM-score closer to 0 means higher deviation. Furthermore, the RMSD score is proportional to the deviation between the wild type and the mutated structures. Finally, the visualization of the wild type and mutants’ structures were performed by Chimera tool [49].

### Structural effect of point mutation *rpoB*-encoded protein

The energy minimization is essential to determine the proper molecular arrangement in space; it used to eliminate high energies in the predicted model and achieve local minima that is closer to native structure [50]. For this reason, the energy minimization was performed for the wild type and mutants’ structure; in addition, the structural consequences of mutations were visualized by Chimera tool [49].

### Prediction of protein-protein interactions

Protein-protein interaction plays key role in predicting the protein function of target protein and drug ability of molecules. The majority of genes and proteins realize resulting phenotype functions as a set of interactions [51]. To investigate the interaction of *rpoB*-encoded protein with various proteins, the STRING database was used (https://string-db.org) [52]. This database aims to integrate all known and predicted associations between proteins, including both physical interactions and functional associations [53].

## Results

### *rpoB* gene’s reference sequence and mutations’ datasets

In order to identify the *rpoB* gene mutations associated with *C. trachomatis* resistance to antibiotics, we performed a literature search pertaining to the topic exhaustively, and a total of nine mutations were retrieved (Table 1). Furthermore, the *rpoB* gene sequence of *C. trachomatis* reference strain BU-434/L2 was extracted from NCBI database using the published accession number AY623623.1 [24].

**Table 1:** Associated *rpoB* mutations with *C. trachomatis* resistance to antibiotics

Author name	Study date	Reference	***C. trachomatis*** strain	Antibiotic	Gene	Mutation	Accession number
Rupp et al.	2007	[20]	BU-434/L2	Rifampin	*rpoB* gene	Q458K H471Y H471N	NC
Suchland et al.	2005	[25]	BU-434/L2	Rifampin	*rpoB* gene	I517M V466A H471L	NC
Suchland et al.	2005	[25]	BU-434/L2	Rifalazil	*rpoB* gene	I517M V466A H471L	NC
Kutlin et al.	2005	[24]	UW-3/Cx/D	Rifampin and rifalazil	*rpoB* gene	H471Y	AY623623
Kutlin et al.	2005	[24]	BU-434/L2	Rifalazil	*rpoB* gene	V136F	AY623623
Dresses-Werringloer et al.	2003	[21]	Serovar K	Rifampin	*rpoB* gene	A467T H471Y	NC

*NC* not cited

### Prediction of *rpoB* mutations’ deleterious effects

According to the rules and recommendation of predictSNP, the results revealed that all the mutations are deleterious. Indeed, the variations V136F, Q458K, A467T, H471Y, H471L, and I517M were shown to be deleterious with a high confidence score 87%, whereas the mutations V466A and H471N were shown to be deleterious with 61% confidence score (Table 2).

**Table 2:** Prediction of mutations’ deleterious effects

SNP	PredictSNP	MAPP	PhD-SNP	PolyPhen-1	PolyPhen-2	SIFT	SNAP	nsSNP-Analyzer	PANTHER
**V136F**	D	D	D	D	D	D	D	D	D
**Q458K**	D	D	D	D	D	D	D	D	-
**V466A**	D	D	D	N	N	D	D	D	N
**A467T**	D	D	D	D	D	D	D	D	N
**H471N**	D	N	D	N	D	D	D	N	N
**H471Y**	D	D	D	D	D	D	D	D	D
**H471L**	D	D	D	D	D	D	D	D	D
**I517M**	D	D	D	D	D	D	D	D	D

N refers to the neutral/stable effect of variant, while D refers to its deleterious/nonstable effect

### Prediction of changes on the protein stability

The eight mutations predicted as deleterious from the previous step were analyzed with both I-Mutant3.0 and MUpro tools; the results showed that the six mutations, V136F, Q458K, V466A, A467T, H471N, and I517M, were predicted to decrease the *rpoB*-encoded protein’s stability (Table 3). Furthermore, the most of the mutations that predicted to decrease the stability of the protein were be shown in *C. trachomatis* strains serovar L2.

**Table 3:** Prediction of changes on the protein stability

SNP	I-Mutant3		MUpro	
	ΔΔG	Prediction	ΔΔG	Prediction
**V136F**	− 1.37	DECREASE	− 0.68	Decrease
**Q458K**	− 0.56	DECREASE	− 1.62	Decrease
**V466A**	− 1.51	DECREASE	− 0.93	Decrease
**A467T**	− 0.84	DECREASE	− 0.9	Decrease
**H471N**	− 0.62	DECREASE	− 0.82	Decrease
**H471Y**	0.27	INCREASE	− 0.36	Decrease
**H471L**	0.52	INCREASE	− 0.05	Decrease
**I517M**	− 1.68	DECREASE	− 0.68	Decrease

### Evolutionary conservation analysis

The six mutations, which were shown decreasing the *rpoB*-encoded protein stability, were analyzed by the ConSurf web server, and the results revealed that these mutations had a high conservation score and located in the highly conserved regions (Table 4); three mutations were predicted as functional and exposed (on protein surface), whereas the rest were predicted to be structural and buried (inside protein core).

**Table 4:** Evolutionary conservancy of amino acids in *rpoB*

Residue and position	Conservation score	Prediction
**V136**	9	Highly conserved and buried (S)
**Q458**	9	Highly conserved and exposed (F)
**V466**	9	Highly conserved and buried (S)
**A467**	8	Highly conserved and exposed (F)
**H471**	9	Highly conserved and exposed (F)
**I517**	9	Highly conserved and buried (S)

### Proteins’ 3D structure modeling and validation

**Fig. 2:**
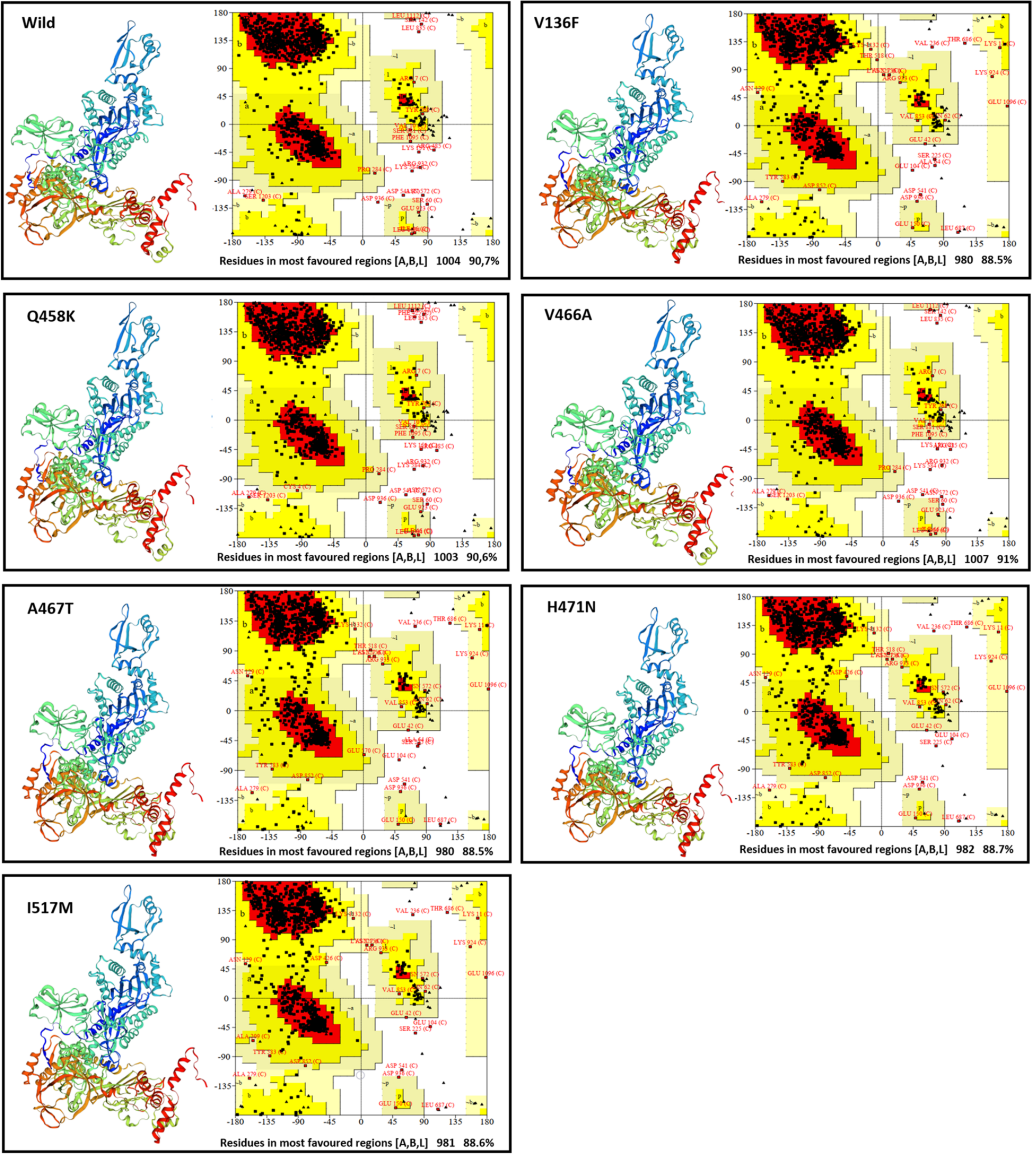
Proteins’ 3D models and Ramachandran plots

The tertiary structures of *rpoB* wild type and mutants’ proteins were subjected to a modeling process using the Swiss-Model (Table 5). Furthermore, the Ramachandran plot of each model shows that the residues in most favored regions are greater than 80%, which explain the accurate of the modeling results (Fig. 2).

**Table 5:** Structural assessment scores

Models	QMEAN score	Sequence identity	Ramachandran favorable region (%)
**Wild**	− 1.69	51.90 %	90.7 %
**V136F**	− 2.34	51.82 %	88.5 %
**Q458K**	− 1.89	51.82 %	90.6 %
**V466A**	− 1.80	51.90 %	91 %
**A467T**	− 2.19	51.82 %	88.5 %
**H471N**	− 2.23	51.82 %	88.6 %
**I517M**	− 2.15	51.82 %	88.7 %

### Structure analysis of the wild type and mutant models

The comparison between mutants and wild type structural 3D models was performed using TM-align; the results showed that the models, A467T, H471N, I517M, and V136F, had the highest root mean square deviation (RMSD = 3.14); furthermore, all the models had template modeling score (TM-score) near to 1; these results signify that the status of protein folding is identical. In addition, the structural 3D models had high RMSD score which signify a high deviation between mutants and wild type (Table 6). Consequently, the 3D mutants’ models, V136F, A467T, H471N, and I517M, were considered for more explorations and further analysis.

**Table 6:** TM-align analysis

SNP	RMSD	Tm score
**V136F**	3.14	0.93293
**Q458K**	0.54	0.99830
**V466A**	0.44	0.99887
**A467T**	3.14	0.93296
**H471N**	3.14	0.93293
**I517M**	3.14	0.93294

### Structural effect of point mutation in *rpoB*-encoded protein

**Fig. 3:**
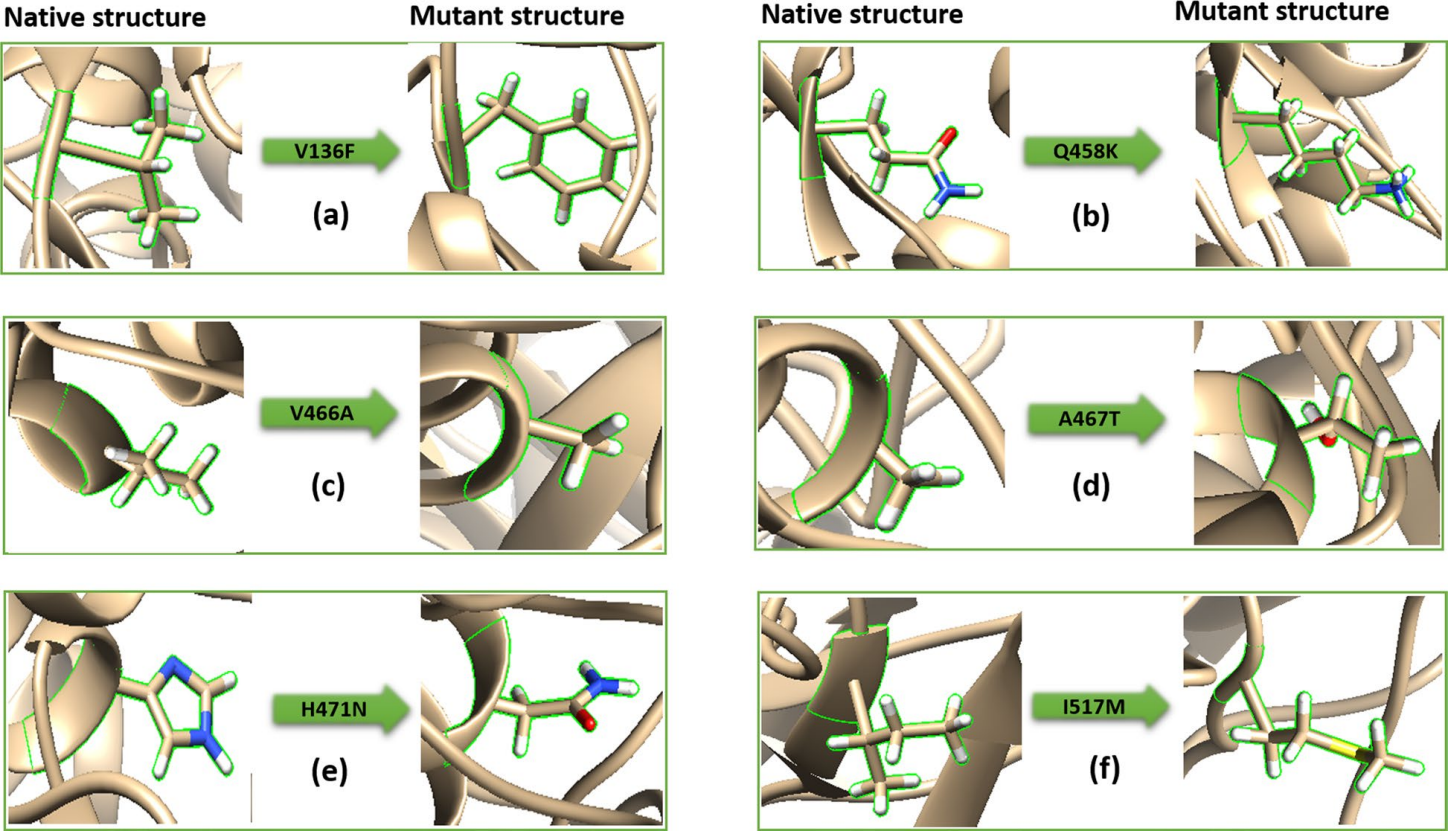
Comparison between native and mutant *rpoB*-encoded proteins’ tridimensional structures. **a** Wild type V and mutant F residues at 136th position (V136F). **b** Wild type Q and mutant K residues at 458th position (Q458K). **c** Wild type V and mutant A residues at 466th position (V466A). **d** Wild type A and mutant T residues at 476th position (A476T). **e** Wild type H and the mutant N residues at 471th position (H471N). **f** Wild type I and mutant M residues at 517th position (I517M)

The energy minimization results showed a wide variance between the energy of wild type and the energy of each mutant’s model (Table 7); in addition, the mutants’ structures have more hydrogen bonds (H-Bond) interactions with the adjacent molecules which signify the dispute between wild type and models energies (Fig. 3).

**Table 7:** Energy minimization

Models	H-Bond	Total energy before energy minimization	Total energy after energy minimization
**Wild**	1054	− 82498.723359	− 103016.025122
**V136F**	1149	− 92419.636836	− 111911.006170
**A467T**	1128	− 90960.932537	− 110710.926310
**471N**	1143	− 93179.859414	− 112836.201011
**I517M**	1137	− 92718.651461	− 112342.326497

### Prediction of protein-protein interactions

**Fig. 4:**
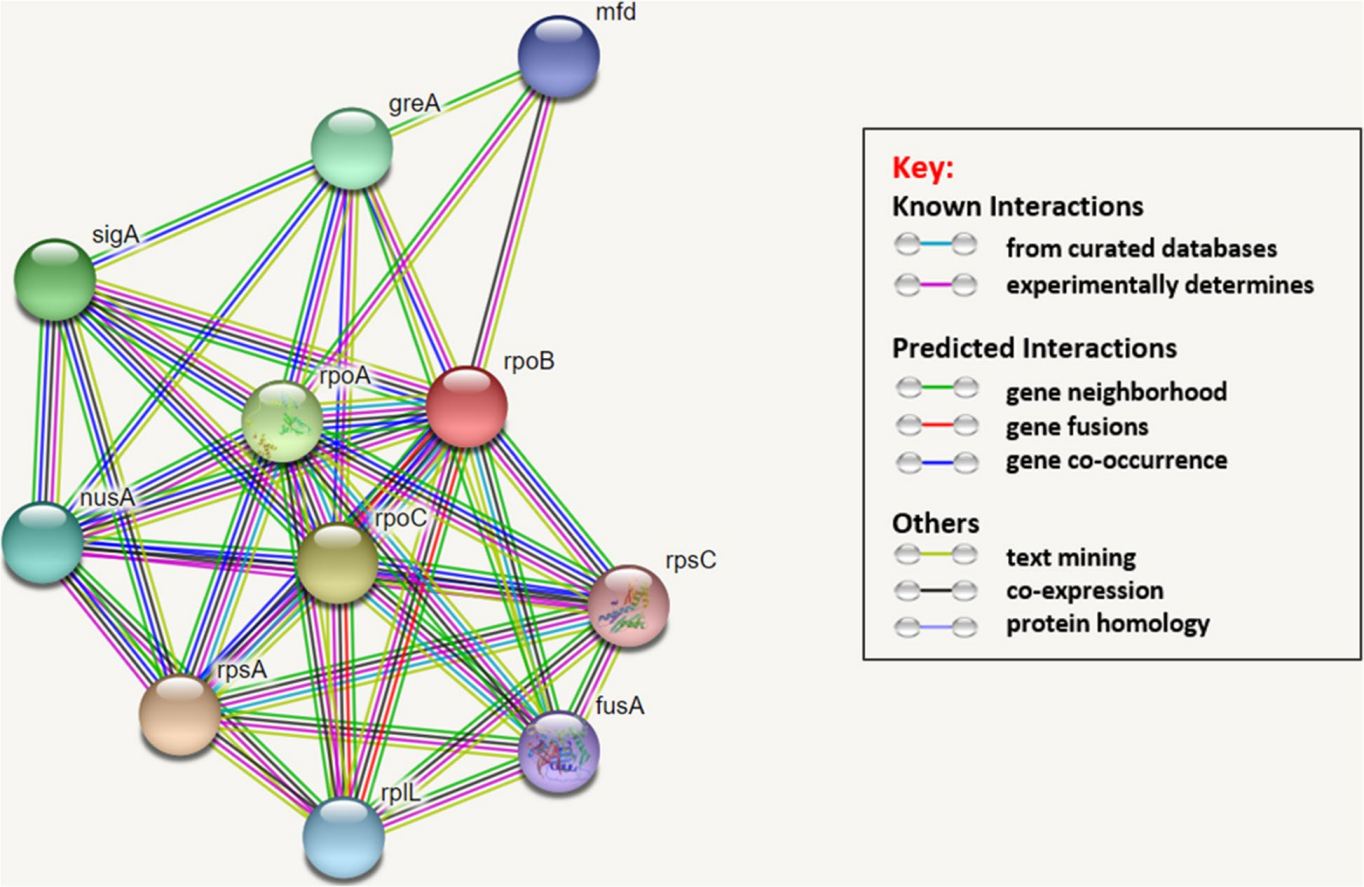
*rpoB* protein-protein interaction network

The prediction of protein-protein interactions using STRING indicated that *rpoB*-encoded protein interacts with 10 proteins from *C. trachomatis* bacteria, including *rpsA*, *rpoC*, *rpoA*, *sigA*, *greA*, *nusA*, *rplL*, *mfd*, *fusA*, and *rpsC* (Fig. 4).

It is known that any occurred changes in the protein can affect the protein network interaction. Therefore, the results revealed that *rpoB* network have high confidence interaction scores; moreover, the molecular action of *rpoB*-encoded protein with other proteins could be modified (Table 8).

**Table 8:** Prediction of molecular interaction of *rpoB* with other proteins

Protein	Information	Score
**rpsA**	Binds mRNA; thus facilitating recognition of the initiation point. It is needed to translate mRNA with a short Shine-Dalgarno (SD) purine-rich sequence (By similarity)	0.999
**rpoC**	DNA-dependent RNA polymerase catalyzes the transcription of DNA into RNA using the four ribonucleoside triphosphates as substrates	0.999
**rpoA**	DNA-dependent RNA polymerase catalyzes the transcription of DNA into RNA using the four ribonucleoside triphosphates as substrates	0.999
**sigA**	Sigma factors are initiation factors that promote the attachment of RNA polymerase to specific initiation sites and are then released. This sigma factor is the primary sigma factor during exponential growth	0.998
**greA**	Necessary for efficient RNA polymerase transcription elongation past template-encoded arresting sites. The arresting sites in DNA have the property of trapping a certain fraction of elongating RNA polymerases that pass through, resulting in locked ternary complexes. Cleavage of the nascent transcript by cleavage factors such as GreA or GreB allows the resumption of elongation from the new 3′ terminus. GreA releases sequences of 2 to 3 nucleotides (By similarity)	0.997
**nusA**	Participates in both transcription termination and antitermination DNA-directed RN polymerase subunit beta; DNA-dependent RNA polymerase catalyzes the transcription of DNA into RNA using the four ribonucleoside triphosphates as substrates	0.995
**rplL**	Forms part of the ribosomal stalk which helps the ribosome interact with GTP-bound translation factors. Is thus essential for accurate translation	0.993
**mfd**	Couples transcription and DNA repair by recognizing RNA polymerase (RNAP) stalled at DNA lesions. Mediates ATP-dependent release of RNAP and its truncated transcript from the DNA, and recruitment of nucleotide excision repair machinery to the damaged site	0.993
**fusA**	Catalyzes the GTP-dependent ribosomal translocation step during translation elongation. During this step, the ribosome changes from the pre-translocational (PRE) to the post-translocational (POST) state as the newly formed A-site-bound peptidyl-tRNA and P-site-bound deacylated tRNA move to the P and E sites, respectively. Catalyzes the coordinated movement of the two tRNA molecules, the mRNA and conformational changes in the ribosome (By similarity)	0.988
**rpsC**	Binds the lower part of the 30S subunit head. Binds mRNA in the 70S ribosome, positioning it for translation	0.987

## Discussion

The treatments adopted commonly against *C. trachomatis* infections are macrolides, tetracyclines, rifamycins, and quinolones [54]. However, the bacteria can acquire resistant to different antibiotics family via a range of mechanisms. The main causes of resistance to antibiotics are: the abusive usage of antibiotics, the spread of resistant strains, or the spread of genes bearing information able to induce resistance [55].

Different studies revealed that the resistance to rifamycin was associated with mutations in the *C. trachomatis* RNA polymerase β-subunit gene (*rpoB*) [20, 21, 24, 25, 56]; these mutations could have a deleterious or neutral impacts on the encoded proteins; moreover, to understand the impact of these mutations on the proteins’ biological functions, stability, and structure, an in silico analysis was performed.

In genomics and proteomics, the in silico analysis plays a significant role to predict the impact of the mutations on the proteins’ function and structure; this analysis can be performed using different bioinformatics tools. However, using these tools could have strengths and weaknesses in the predictions, because every algorithm uses different parameters for prediction [57, 58]. Consequently, to screen and prioritize the candidate functional SNPs requires the implementation of algorithms with different parameters and aspects to combine their advantages, enhance the accuracy and reliability of the predictions, and minimize the errors [59–61]. Basically, to perform the SNP prediction, it is recommended to use at least five tools to obtain an agreement on the effect of the variations on the structure and function of the studied proteins [58]. In our approach, nine tools were used to predict the SNP deleterious effects on function and structure of the *rpoB*-encoded protein (Fig. 1); this protocol was adopted by different investigators [62–64]. Our results revealed that the nine used tools showed that all the SNPs (*n* = 8) have a deleterious effect on the encoded protein (Table 2); indeed, almost the same results of prediction were found by the different used tools; this explains the prediction accuracy and result validity.

It is known that the protein structure governs its stability and determines its function [65]; in our study, the prediction of changes on the proteins’ stability showed differences in the predictions’ results (Table 3). Indeed, the stability prediction using I-Mutant3.0 and MUpro showed discrepant results for the mutations H471Y and H471L, which consist in decreased proteins’ stability by MUpro, and increased proteins’ stability by I-Mutant3.0. In addition, according to the retrieved results, the six mutations, V136F, Q458K, V466A, A467T, H471N, and I517M, were shown to be destabilizing the proteins’ structure by indicating a negative score for the Gibbs free energy. Hence, these mutations can cause misfolding, degradation, or aberrant conglomeration of the *rpoB*-encoded proteins. The results discrepancies’ can be considered as a negative aspect of the analysis; for that, we suggest performing deeper prediction using new tools.

On the other hand, the ConSurf results revealed that the studied mutations had high conservation scores and located in the highly conserved regions (Table 4). The mutation position can directly affect the proteins’ function and structure; consequently, the mutations position could affect the drug accessibility to the bacteria and so its level of antibiotics sensitivity.

The wild protein 3D structures are essential to more understand the functional and structural effect of mutations; the *rpoB* protein structure was not available in the Protein Data Bank (PDB). Thus, we predict the 3D structure of wild type and mutants, by changing the six mutations into the native sequence. In addition, Ramachandran plot analysis was performed to validate these protein structures; all the structures had the residues in most favored regions more than 80%, which means that all the structures are valid (Fig. 2). Hence, no negative aspect was notified using the Swiss-Model Server.

Additionally, the structural changes in the encoded proteins were analyzed using TM-Align to compute the RMSD and the TM-score by superimposing models of native and mutant proteins. The results showed that the models with the mutations V136F, A467T, H471N, and I517M had high RMSD values and their TM-score near to 1 (Table 6). Hence, these results indicate a quite large structural dissimilarity between the native and mutant models. Indeed, the structural changes of mutants’ proteins indicate potential alterations in the binding affinity of mutants’ structures of *rpoB*-encoded proteins with their receptors which may lead to resistance to antibiotics. Regarding the predictions of the mutations Q458K and V466A, the results showed a lowest RMSD score and their TM-score near to 1; these results signify that the status of the protein folding is identical.

In another hand of the analysis, the results of the energy minimization revealed that the total energy of the mutants: V136F, A467T, H471N, and I517M were low compared to that of the wild type protein. Moreover, the mutants present the higher number of H-bonds. In opposition to the high total energy, the wild type structure had the lower number of H-bonds (Table 7). The discrepancy of results found between wild type and mutant structures signifies the dispute between wild type and mutants’ models, which can explain the observed resistance to antibiotics mechanisms.

At the final step of our in silico analysis, the prediction of the protein-protein interaction revealed that *rpoB* interacts with 10 different bacteria’s proteins (Fig. 4), which are showed playing a role in the bacteria cycle progression and DNA replication events (Table 8). In addition, STRING predicts these protein-protein interactions with high confidence scores; therefore, it can be suggested that any change in the *rpoB*-encoded protein structure and function might affect the bacteria sensitivity to antibiotics.

## Conclusion

This study compiled the mutations in *rpoB* gene which were revealed to be associated with *C. trachomatis* resistance to rifamycin and predicts their effects using various bioinformatics tools. The results revealed that the mutations, V136F, A467T, H471N, and I517M, had the most impact on both stability and RMSD, in addition to their location in the highly conserved regions; therefore, they can affect the protein’s function, structure, and their interaction with other proteins. All these changes can explain the observed resistance to antibiotics. Moreover, the study revealed that all mutations are not necessarily translated to strong phenotypic expression. Consequently, the revealed information through this in silico analysis would be useful for deeper exploration to understand the mechanisms of *C. trachomatis* resistance; this could enable managing the infection and avoid its complications. We recommend further investigations to perform deeper experimental analysis and explore alternative therapies and new drug design by molecular docking; this can be done in collaboration between bioinformaticians, physicians, biologists, pharmacists, and chemistry and biochemistry scientists.

## Data Availability

Not applicable.

## Abbreviations

*C. trachomatis*: *Chlamydia trachomatis*
*Ca*: *Candidatus*
SNP: Single nucleotide polymorphism
ΔG: Gibbs free energy of unfolding
ΔΔG: Change in Gibbs free energy
SVM: Support vector machine
TM-score: Template modeling score
RMSD: Root means square deviation
BLAST: Basic Local Alignment Search Tool
PSI-BLAST: Position-Specific Iterative Basic Local Alignment Search Tool
QMEAN: Qualitative Model Energy Analysis
NCBI: National Center for Biotechnology Information
H-Bond: Hydrogen bond
